# A deep learning model for brain segmentation across pediatric and adult populations

**DOI:** 10.1038/s41598-024-61798-6

**Published:** 2024-05-22

**Authors:** Jaime Simarro, Maria Ines Meyer, Simon Van Eyndhoven, Thanh Vân Phan, Thibo Billiet, Diana M. Sima, Els Ortibus

**Affiliations:** 1 icometrix, Leuven, Belgium; 2 Department of Development and Regeneration, KU Leuven, Leuven, Belgium; 3 Department of Pediatric Neurology, UZ Leuven, Leuven, Belgium; 4 Child and Youth Institute, KU Leuven, Leuven, Belgium

**Keywords:** Neurology, Neurodegenerative diseases, Neurodevelopmental disorders, Paediatric neurological disorders, Biomedical engineering

## Abstract

Automated quantification of brain tissues on MR images has greatly contributed to the diagnosis and follow-up of neurological pathologies across various life stages. However, existing solutions are specifically designed for certain age ranges, limiting their applicability in monitoring brain development from infancy to late adulthood. This retrospective study aims to develop and validate a brain segmentation model across pediatric and adult populations. First, we trained a deep learning model to segment tissues and brain structures using T1-weighted MR images from 390 patients (age range: 2–81 years) across four different datasets. Subsequently, the model was validated on a cohort of 280 patients from six distinct test datasets (age range: 4–90 years). In the initial experiment, the proposed deep learning-based pipeline, icobrain-dl, demonstrated segmentation accuracy comparable to both pediatric and adult-specific models across diverse age groups. Subsequently, we evaluated intra- and inter-scanner variability in measurements of various tissues and structures in both pediatric and adult populations computed by icobrain-dl. Results demonstrated significantly higher reproducibility compared to similar brain quantification tools, including childmetrix, FastSurfer, and the medical device icobrain v5.9 (*p*-value< 0.01). Finally, we explored the potential clinical applications of icobrain-dl measurements in diagnosing pediatric patients with Cerebral Visual Impairment and adult patients with Alzheimer’s Disease.

## Introduction

Neuroimaging techniques play a crucial role in advancing our understanding of the human brain, covering its structure, development, function, and pathologies^1^. Magnetic Resonance Imaging (MRI) stands out as a non-invasive technology to obtain high-resolution, in vivo measurements of the human brain^2^. Automated analysis of MR images contributes to the diagnosis of neurological pathologies across various life stages, from childhood (e.g., focal cortical dysplasia^3^) to late adulthood (e.g., Alzheimer’s disease^4^).

Quantitative assessment, exemplified by volumetric analysis, enhances the objectivity of brain interpretation compared to visual MRI scan inspection alone. Traditional techniques for brain MR image segmentation involve atlas-based methods and statistical models, such as FreeSurfer^5^, volBrain^6^, or the medical device software, icobrain v5.9^7,8^. Nevertheless, recent progress in deep learning models, such as QuickNat^9^, AssemblyNet^10^, and FastSurfer^11^, has demonstrated superior performance compared to traditional methodologies, as evidenced in a recent review^12^.

Despite the growing role of quantitative analysis tools, additional technical and clinical validation is required^4^. Notably, there is a lack of validated models for robust and reliable brain quantification in multi-scanner settings, common in clinical data. Additionally, recent algorithms, including deep learning methods, are usually developed and validated using adult datasets. However, standard MRI processing methods designed for adult images may not be suitable for pediatric datasets^13^. Pediatric brain analysis poses unique challenges such as reduced tissue contrast, within-tissue intensity heterogeneities, and smaller regions of interest^13,14^. Consequently, pediatric brain analysis commonly employs specialized analysis tools like childmetrix^15^.

In pediatric studies, a common dilemma arises regarding the use of age-appropriate methods for different developmental stages or maintaining a consistent method across all ages^16^. While age-specific models are optimized for specific age ranges, their use introduces the risk of attributing age-related differences to methodological inconsistencies rather than genuine brain development or change. Particularly when monitoring patients across different transitional phases, such as from the pediatric stage through adolescence and into adulthood, there is a significant need for a general, consistent, and reliable method, eliminating reliance on multiple age-specific methods.

In this work, we develop and validate a brain segmentation pipeline across pediatric and adult populations, emphasizing the impact of heterogeneous and representative training data rather than the optimization of the deep learning architecture employed. The primary objective of this study is to explore whether a single deep learning model can be optimized to consistently quantify structural MRI across the lifespan, reflecting the distinctive neuroanatomy of each developmental stage. We hypothesize that a single deep learning model trained on datasets covering a wide age range will perform comparably to age-specific models within their respective age groups. The secondary objective is to validate the proposed pipeline’s performance in terms of reproducibility, diagnostic accuracy, and computational time. We hypothesize that the proposed deep learning-based pipeline will produce results comparable to established methods such as childmetrix, icobrain v5.9, and FastSurfer, while ensuring accurate and reproducible brain quantification across pediatric and adult populations.

## Materials and methods

### Datasets

Four separate datasets collectively containing 390 patients, aged between 2 and 81 years, were utilized for training. Validation was performed on a separate cohort of 280 patients from six distinct test datasets, covering an age range from 4 to 90 years. These datasets consisted of 757 T1-weighted MRI scans acquired from various manufacturers (Philips, Siemens, GE, Fujifilm) with different magnetic field strengths (1.5T/3T ∼ 32%/68%) across 21 scanners. The patients represented a diverse pathological conditions, including developmental disorders, cerebral visual impairment, depression, bipolar disorder, schizophrenia, multiple sclerosis, and Alzheimer’s disease. Table 1 presents a summary of the diverse datasets employed in this retrospective study. Further details about these datasets can be found in Appendix A.

**Table 1:** The datasets utilized for model training and validation consisted of both pediatric (denoted with suffix p) and adult (denoted with suffix a) data.

Scenario	Dataset	Subjects	Age range (years)	Diagnosis	Female (%)	Scanner type	Source
Training	1.1.p	157	5–21	DD and HC	40%	3T: Siemens	CMI-HBN^17^
	1.2.p	66	2–6	HC	48%	3T: GE	Calgary^18^
	1.3.a	32	Adults	VLOSLP, LOD and HC	NA	3T: Philips	Research cohort^19^
	1.4.a	135	16–81	MS	60%	1.5–3T: Siemens, GE and Philips, 1.5T: Fujifilm	Clinical practice
Accuracy	2.p	103	4–16	BD, Sz and HC	45%	1.5T: GE	CANDIShare^20^
	2.a	30	18–90	HC	66%	1.5T: Siemens	MICCAI2012^21^
Reproducibility	3.p	70	6–17	DD and HC	44%	3T: Siemens	NKI^22^
	3.a	10	39–57	MS	70%	3T: Siemens, GE and Philips	Re3T^7^
Diagnosis Performance	4.p	21	4–13	CVI and no CVI	23%	1.5T:Siemens and Philips, 3T: Philips	Clinical practice
	4.a	46	58–85	AD and HC	54%	1.5T: GE	MIRIAD^23^

A subset of patients were randomly selected from original training datasets. These datasets include individuals with Developmental Disorders (DD), Healthy Control (HC), Very-Late-Onset Schizophrenia-Like Psychosis (VLOSLP), Late-Onset Depression (LOD), Bipolar Disorder (BD), Schizophrenia (Sz) and Multiple Sclerosis (MS), Cerebral Visual Impairment (CVI) and Alzheimer’s Disease (AD). NA = not available.

#### Training dataset

The training dataset comprises a wide age range, pathologies and acquisition protocols. T1-weighted images were sourced from pediatric datasets, including the Healthy Brain Network (HBN, dataset 1.1.p)^17^ and the Calgary Preschool MRI (dataset 1.2.p)^18^. Additionally, T1-weighted images of adult patients were obtained from a research cohort (dataset 1.3.a) focused on the relations between very-late-onset schizophrenia-like psychosis, hippocampal volume, early adversity, and memory function^19^ as well as another cohort from clinical practice (dataset 1.4.a).

#### Segmentation accuracy testing dataset

Two publicly available manually annotated datasets were used to validate the segmentation accuracy: the Child and Adolescent NeuroDevelopment Initiative (CANDI, dataset 2.p)^20^ and the MICCAI 2012 Grand Challenge and Workshop on Multi-Atlas Labeling (MICCAI2012, dataset 2.a)^21^. We excluded 5 images from the latter due to repeated scans of the same patient.

#### Reproducibility testing dataset

The reproducibility of the measurements was evaluated by analyzing two images from the same individual acquired with re-positioning within a very short time interval, ensuring no anatomical change between the two images (i.e., test and retest images). Two test-retest datasets were used to validate the reproducibility. The first dataset is a pediatric intra-scanner dataset obtained from Nathan Kline Institute (NKI, dataset 3.p)^22^, while the second dataset comprises 10 adult individuals who underwent two scans, using three different types of scanners (Re3T, dataset 3.a)^7^. Using repeated scans in multiple scanner types enables analysis for intra-scanner and inter-scanner validation.

#### Diagnostic performance testing dataset

The diagnostic performance is assessed using two separate datasets. The first dataset comprises pediatric patients suspected of suffering from Cerebral Visual Impairment (CVI) (dataset 4.p), approved by the local Ethical Committee of UZ Leuven, Belgium (S65276). All methods were carried out in accordance with relevant guidelines and regulations. Informed consent was obtained from all subjects or their legal guardians. Secondly, we used the Minimal Interval Resonance Imaging in Alzheimer’s Disease (MIRIAD, dataset 4.a), which includes both patients with Alzheimer’s Disease (AD) and healthy elderly individuals^23^.

### icobrain-dl pipeline: design and development

icobrain-dl is a pipeline for brain quantification. The pipeline processes a 3D T1-weighted MR image as input and undergoes three main steps: preprocessing, brain segmentation using a deep learning model, and brain quantification. The output includes brain segmentation masks for various regions of interest (ROIs) and brain volumes.

#### Pre-processing

Prior to training, the images underwent several fully automated pre-processing steps. Firstly, bias-field correction was performed using the N4 inhomogeneity correction algorithm as implemented in the Advanced Normalization Tools (ANTs) toolkit^24^. In pediatric cases, an age-specific atlas is used to obtain the brain mask for N4 correction. Secondly, the images were affinely registered to MNI space using the $\texttt {reg\_aladin}$ algorithm in NiftyReg^25^. To minimize the effect of outliers, intensities were clipped at the 1^st^ and 99^th^ percentile. Finally, the intensities were normalized using a variation on z-scoring, this function was computed over values above the 10^th^ percentile, with preference given to the median over the mean. The standard deviation was then computed within the 90^th^ percentile.

#### Simultaneous segmentation of brain tissue and structures via a multi-head deep learning model

The proposed deep learning model is designed to perform two tasks, brain tissue segmentation and brain structural segmentation, whose labels are not mutually exclusive.

- Task 1: Tissue segmentation. This task involves the segmentation of brain tissues into four distinct classes: background (i.e., not brain tissue), white matter (WM), gray matter (GM), and cerebrospinal fluid (CSF).
- Task 2: Structural segmentation. This task involves the segmentation of 22 anatomical brain structures and background. A detailed list of the structures is provided in Appendix B.

**Figure 1:**
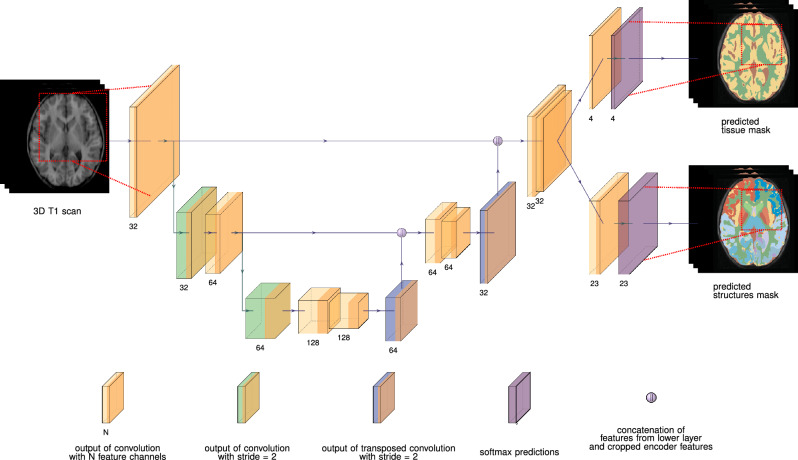
The deep learning model processes a 3D T1-weighted image via a single-input, dual-output 3D convolutional neural network (CNN) to produce estimated multi-label masks for brain tissues (background, white matter, gray matter, cerebrospinal fluid) and brain structures (background + 22 brain structures). The CNN is based on the widely used 3D U-net architecture, which operates on 3D patches of the input scan. Each convolutional layer utilizes $3\times 3\times 3$ kernels, except for the two convolutional layers before the softmax layers, which use $1\times 1\times 1$ kernels. Weight normalization and leaky ReLU (slope = 0.20) are employed. The output patches have dimensions of $88\times 88\times 88$ voxels, which are smaller than the input patches’ dimensions ($128\times 128\times 128$ voxels) due to the use of valid convolutions, mitigating off-patch-center bias.

The architecture utilizes a 3D U-net backbone^26^, incorporating two segmentation heads. Each of both outputs is a softmax array of $N_k$ probability maps, where $N_k$ is the number of classes being predicted in task *k*. Moreover, certain modifications were made to the original architecture, including substituting batch normalization with weight normalization^27^, using leaky ReLU as the primary activation function, and using strided convolutions instead of max pooling^28^. Figure 1 illustrates our final architecture, while detailed information including justification for the multi-task architecture can be accessed in Appendix C.

The model was trained using a weighted sum of the per-task losses, each comprising of a soft Dice loss ($L_{Dice}$) and a weighted categorical cross-entropy loss ($L_{\text {w}CE}$), as shown in Eq. (1).

1 $$\begin{aligned} \mathscr {L}_{total} = ~ \alpha _1\left( \mathscr {L}^{(1)}_{\text {w}CE} + \mathscr {L}^{(1)}_{Dice}\right) +~ \alpha _2\left( \mathscr {L}^{(2)}_{\text {w}CE} + \mathscr {L}^{(2)}_{Dice}\right) \end{aligned}$$

We set $\alpha _1=1$ (tissue segmentation) and $\alpha _2=10$ (structural segmentation).

The proposed model is trained on patches of $128\times 128\times 128$ voxels from T1-weighted MR images acquired without contrast agent injection. To augment the variability in the training set, ensuring that the range of intensities and tissue contrasts is similar with those observed in multi-center, multi-scanner cohorts, we applied intensity-based data augmentation as described in Meyer et al.^29^. This technique uses Gaussian Mixture Modeling to change the intensity of the individual tissue components within an MR image while preserving structural information. We utilized the predefined default parameters of the public implementation of this code, available at https://github.com/icometrix/gmm-augmentation.

The model was implemented using Tensorflow 2.6 and employed He weight initialization. The training process was stopped upon detecting convergence of the validation loss. The validation set, which constituted a randomly selected 15% of the training dataset, was not utilized for optimizing the network weights. Adam optimizer was deployed with an initial learning rate of $\lambda = 0.001$.

#### Efficient generation of high-quality training labels

To address the challenge of obtaining manual annotations for large datasets, we created ‘silver’ ground truth, starting from the labels predicted by icobrain v5.9 on the training datasets. Subsequently, minor manual corrections were made where necessary.

#### Models training scheme

We trained three deep learning models with identical architecture, each using a different set of data for training:

- The *icobrain-dl* model was trained on both pediatric and adult data, providing the most comprehensive training dataset (i.e., datasets 1.1.p, 1.2.p, 1.3.a and 1.4.a ).
- The pediatric-specific model, termed *icobrain-dl-p*, was exclusively trained on pediatric datasets (i.e., datasets 1.1.p and 1.2.p).
- The adult-specific model, termed *icobrain-dl-a*, was solely trained on adult datasets (i.e., datasets 1.3.a and 1.4.a ).

### Validating technical and diagnostic performance

Two sets of experiments were conducted to validate both technical and diagnostic performance, with a focus on segmentation accuracy, intra- and inter-scanner variability, and computational time.

Segmentation accuracy was evaluated through the Dice similarity coefficient (DSC) and Hausdorff distance (HD)^30^. DSC is a metric quantifying the overlap between two segmentation masks, with values ranging from 0 (indicating no overlap) to 100 (indicating perfect agreement). The HD measures the maximal contour distance (in millimeters) between the two masks. A smaller HD indicates greater similarity between the masks. To address the high sensitivity of the HD to outliers^31^, we considered the 95th percentile of the HD, denoted as HD95. In the initial experiment, DSC and HD95 calculations were performed between ground truth segmentations and both icobrain-dl and the age-specific models (icobrain-dl-p or icobrain-dl-a). Subsequently, DSC and HD95 values were computed between the icobrain-dl model and the age-specific models on datasets 2.p and 2.a.

The reproducibility of icobrain-dl was assessed by comparing it with established non-deep learning algorithms, specifically the pediatric-focused *childmetrix*^15^ and the clinically-used adult-focused medical device software icobrain v5.9, referred to as *icobrain-nondl*^7,8^. Additionally, the state-of-the-art deep learning model *FastSurfer*^11^ was included. Test-retest relative differences were computed with respect to the mean volumes across methods (dataset 3.p and 3.a), and the Wilcoxon signed-rank test was employed to identify significant differences between methods at levels of 0.01 and 0.001.

The validation of diagnostic performance serves as a proof of concept for the clinical application of the segmentation algorithm. To demonstrate the icobrain-dl’s applicability across both pediatric and adult populations, two pathologies with distinct volumetric patterns were selected. In the first experiment, the objective was to differentiate patients with CVI from those without CVI using the whole brain white matter volume (dataset 4.p), motivated by the known association between periventricular white matter damage and CVI^32^. The second experiment aimed to distinguish patients with AD from cognitively healthy individuals using temporal lobe cortical gray matter volume (dataset 4.a). Previous research has established the reliability of this region in discerning between AD patients and healthy controls^8^. Volumes from the different pipelines were normalized for head size employing the determinant of the affine transformation to the MNI atlas as a scaling factor. Head size-normalized volumes of the regions of interest (i.e., whole brain white matter and temporal lobe cortical gray matter) were used to distinguish pathology and non-pathology. Model comparisons were conducted using the area under the receiver operating characteristic curve (AUC) and the DeLong test, with a significance level of 0.05^33^. The assessment of accuracy, specificity, and sensitivity metrics was based on the maximum value of the Youden index.

## Results

### Accuracy

On the pediatric dataset 2.p, the deep learning models icobrain-dl and icobrain-dl-p exhibited comparable performance in accurately segmenting brain structures, achieving an average DSC of 82.2% and 80.8%, respectively. Their average HD95 were 3.26mm and 3.23mm. Additionally, there was a high overlap between the segmentations of icobrain-dl and the pediatric-oriented icobrain-dl-p, with an average DSC of 87.4% and HD95 1.76mm. Similar results were observed in the adult dataset 2.a, where icobrain-dl achieved an average DSC of 82.6% and HD95 of 2.27mm when compared to manual segmentations. For icobrain-dl-a, the metrics were 81.9% and 2.37mm, respectively. The average DSC between both segmentation models was 92.4% with an average HD95 of 1.02mm. Table 2 and Table 3 display the DSC and HD95 between manual ground truth segmentations and segmentations calculated by the three deep learning models.

These findings suggest that icobrain-dl is as effective as the age-specific models in accurately segmenting brain structures in both pediatric and adult populations.

**Table 2:** icobrain-dl consistently achieves high overlap in segmenting different brain structures across subject age ranges, while only minimally sacrificing accuracy and sometimes even outperforming models that are tailored for specific age ranges (icobrain-dl-p for pediatric data and icobrain-dl-a for adult data).

Dice similarity coefficient (DSC)						
	Pediatric dataset 2.p			Adult dataset 2.a		
	GT vs		icobrain-dl-p vs	GT vs		icobrain-dl-a vs
	icobrain-dl	icobrain-dl-p	icobrain-dl	icobrain-dl	icobrain-dl-a	icobrain-dl
WM	84.8 (2.0)	85.0 (2.1)	92.3 (2.9)	88.5 (1.1)	85.5 (1.0)	91.8 (1.7)
CGM	83.0 (2.5)	79.7 (3.7)	88.2 (3.2)	83.2 (1.6)	82.5 (1.6)	89.9 (2.3)
Lateral ventricles	83.0 (4.6)	83.4 (4.7)	88.5 (6.4)	88.7 (3.6)	88.9 (3.7)	93.3 (2.7)
Hippocampus	78.8 (2.7)	71.4 (11.3)	82.1 (11.3)	76.7 (1.8)	77.2 (1.9)	91.3 (1.7)
Caudate	83.5 (3.5)	82.5 (8.3)	83.5 (9.3)	81.8 (3.1)	80.5 (3.2)	91.5 (2.2)
Putamen	84.8 (1.5)	83.9 (3.1)	89.8 (3.6)	83.4 (2.4)	80.7 (1.8)	92.1 (1.6)
Cerebellar GM	83.9 (2.5)	86.9 (1.9)	89.9 (1.9)	78.4 (2.8)	79.5 (2.4)	94.1 (1.3)
Cerebellar WM	76.1 (4.1)	75.9 (4.4)	81.1 (2.8)	77.8 (2.8)	77.4 (2.3)	92.4 (1.6)
Thalamus	82.6 (2.4)	78.7 (7.0)	90.7 (6.7)	85.0 (1.0)	84.5 (1.3)	94.7 (1.0)

We compare segmentation accuracy as measured by the Dice similarity coefficient expressed as a percentage between three deep learning models that are trained using different subsets of training data against manually created ground truth (GT) from pediatric (CANDIShare, dataset 2.p) and adult (MICCAI2012, dataset 2.a) data. The Dice similarity coefficient between the models’ predictions is also shown. The Dice similarity coefficient is reported as: mean value (standard deviation) across subjects. * WM* = White Matter, * CGM* = Cortical Gray Matter, *GM* = Gray Matter.

**Table 3:** Summary of the Hausdorff distance 95th percentile (HD95) between ground truth (GT) and icobrain-dl or the age-specific models, and between the age-specific models and icobrain-dl.

Hausdorff distance 95th percentile (HD95)						
	Pediatric dataset 2.p			Adult dataset 2.a		
	GT vs		icobrain-dl-p vs	GT vs		icobrain-dl-a vs
	icobrain-dl	icobrain-dl-p	icobrain-dl	icobrain-dl	icobrain-dl-a	icobrain-dl
WM	2.07 (0.52)	2.08 (0.51)	1.11 (0.21)	1.34 (0.24)	1.80 (0.36)	1.01 (0.07)
CGM	2.50 (0.42)	2.81 (0.50)	1.23 (0.35)	1.36 (0.17)	1.38 (0.20)	1.00 (0.00)
Lateral ventricles	6.82 (9.47)	3.73 (6.59)	1.62 (1.67)	1.06 (0.14)	1.06 (0.14)	1.00 (0.00)
Hippocampus	2.50 (0.59)	3.43 (1.67)	1.79 (1.38)	1.93 (0.24)	1.95 (0.27)	1.00 (0.00)
Caudate	1.64 (0.37)	1.86 (1.49)	1.77 (1.28)	1.51 (0.21)	1.64 (0.25)	1.04 (0.12)
Putamen	1.80 (0.27)	2.05 (0.38)	1.37 (0.44)	1.52 (0.23)	1.74 (0.19)	1.01 (0.07)
Cerebellar GM	3.29 (0.47)	3.01 (0.57)	2.13 (0.37)	3.00 (0.25)	3.03 (0.22)	1.04 (0.12)
Cerebellar WM	5.74 (1.40)	6.72 (2.20)	3.24 (1.09)	6.51 (1.43)	6.41 (0.92)	1.16 (0.36)
Thalamus	2.99 (0.37)	3.38 (0.77)	1.65 (1.01)	2.24 (0.09)	2.36 (0.20)	1.00 (0.00)

icobrain-dl consistently achieves high-quality segmentation of various brain structures across different age groups, as indicated by the low HD95 with comparing with GT in both datasets. The HD95 is reported as: mean value (standard deviation) in millimeters across subjects. *WM* = White Matter, *CGM* = Cortical Gray Matter, *GM =* Gray Matter.

### Reproducibility

**Figure 2:**
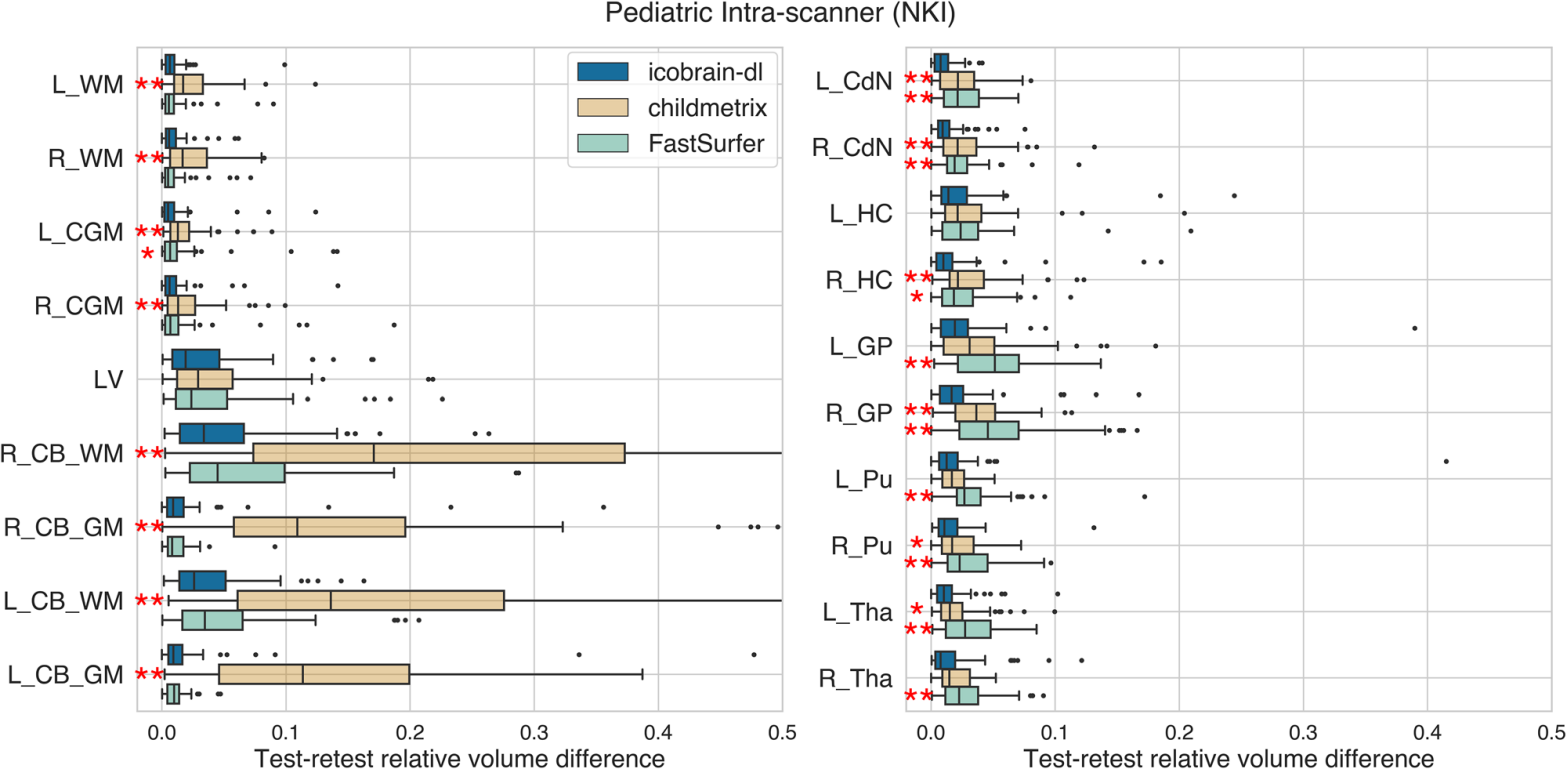
The icobrain-dl measurements exhibited statistically significantly lower test-retest errors than both childmetrix and FastSurfer across a majority of regions for pediatric cases (dataset 3.p) in intra-scanner settings, as quantified by relative test-retest volume differences. Legend: * = $p < 0.01$, **= $p < 0.001$ according to Wilcoxon signed-rank tests comparing icobrain-dl to either childmetrix or FastSurfer. To ensure overall figure readability, certain boxplots have been cropped. * L* = left, *R =* right, *WM =* white matter, *CGM* = cortical gray matter, *LV* = lateral ventricles, *CB* = cerebellum, *CdN* = caudate nucleus, *HC* = hippocampus, *GP* = globus pallidus, *Pu* = putamen, *Tha* = thalamus.

The segmentations generated by icobrain-dl systematically had lower test-retest volume differences for the pediatric intra-scanner setting (dataset 3.p) than childmetrix and FastSurfer, as illustrated in Figure 2. For most structures, these test-retest differences from icobrain-dl were significantly lower than the comparable methods ($p < 0.01$).

**Figure 3:**
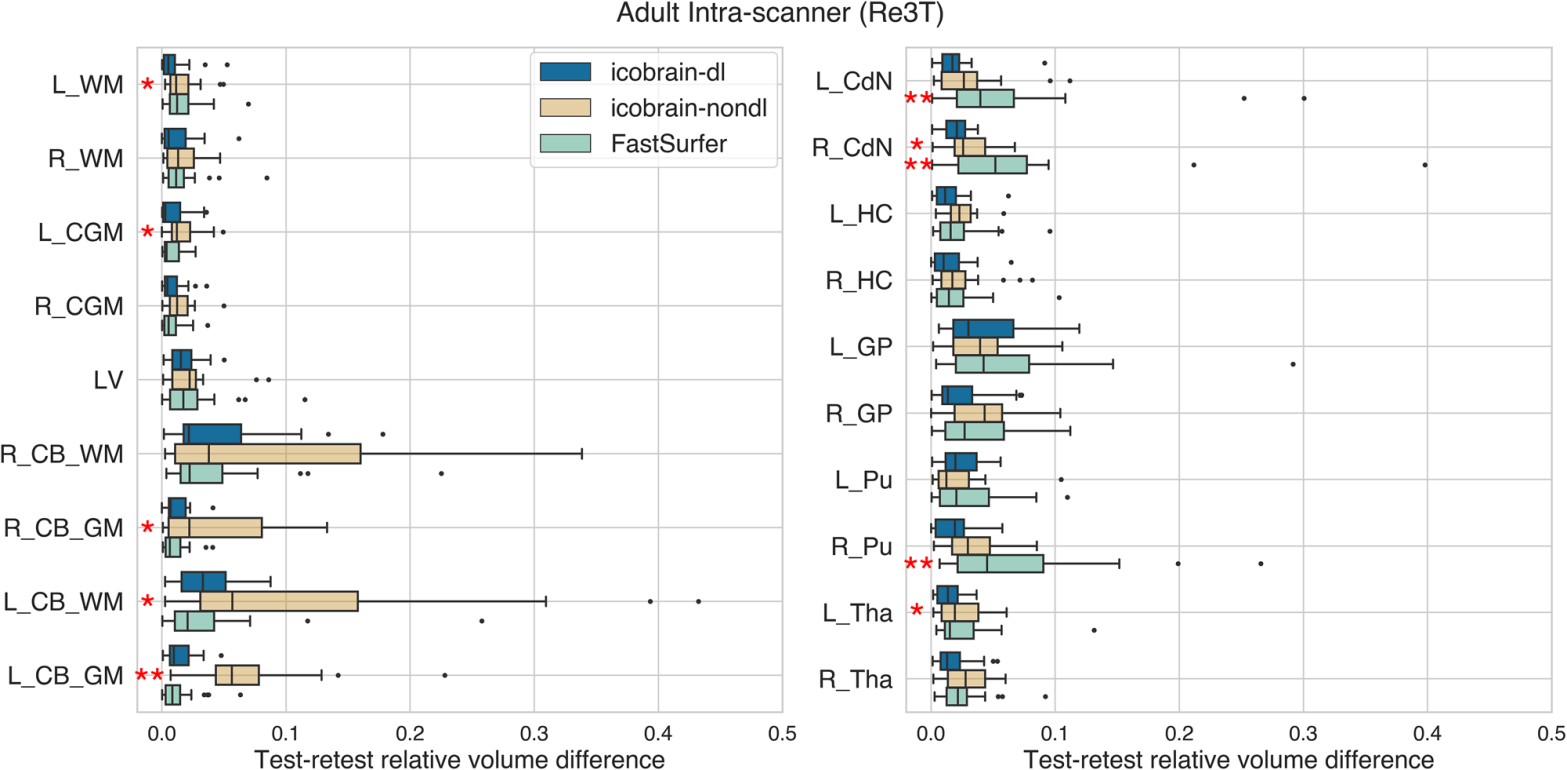
The icobrain-dl measurements exhibited equal or lower test-retest errors than icobrain-nondl and FastSurfer for adult cases in the intra-scanner settings, as quantified by intra-scanner relative test-retest volume differences. Legend: * = $p < 0.01$, **= $p < 0.001$ according to Wilcoxon signed-rank tests comparing icobrain-dl to either icobrain-nondl or FastSurfer. * L* =  left, *R* = right, *WM* = white matter, *CGM* = cortical gray matter, *LV* = lateral ventricles, *CB* = cerebellum, *CdN* = caudate nucleus, *HC* = hippocampus, *GP* =  globus pallidus, *Pu* = putamen, *Tha* = thalamus.

**Figure 4:**
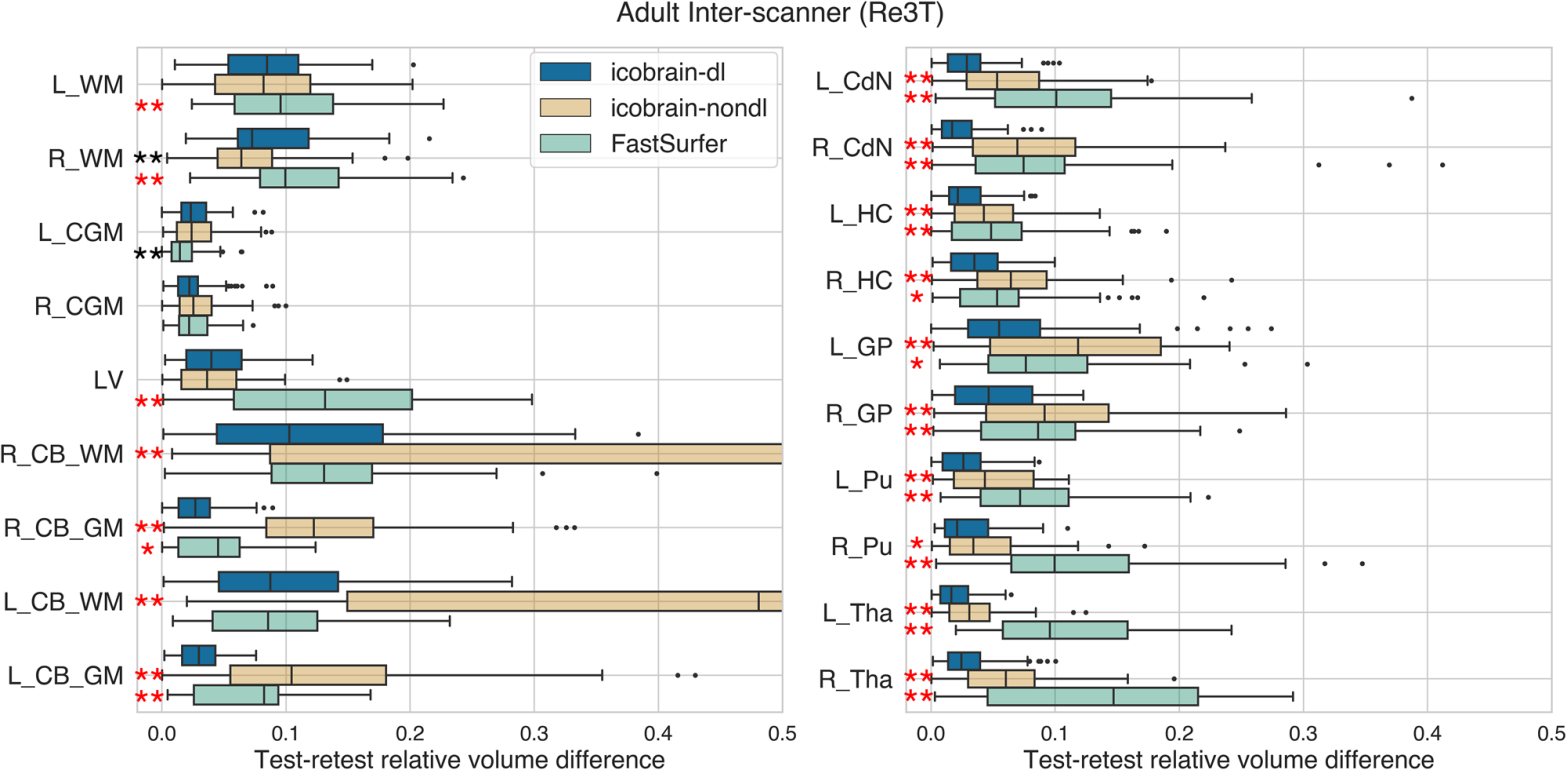
The icobrain-dl measurements exhibited statistically significantly lower test-retest errors than icobrain-nondl and FastSurfer across all the subcortical structures (right) for adult cases (dataset 3.a) in inter-scanner settings, as quantified by relative test-retest volume differences. The asterisk colour indicates the better performing method (red = icobrain-dl, black = state-of-the-art). To ensure overall figure readability, certain boxplots have been cropped. * L* =  left, *R* = right, *WM* = white matter, *CGM* = cortical gray matter, *LV* = lateral ventricles, *CB =* cerebellum, *CdN =* caudate nucleus, *HC =* hippocampus, *GP* = globus pallidus, *Pu =* putamen, *Tha =* thalamus.

A similar pattern of lower test-retest volume differences provided by icobrain-dl was observed in adults (dataset 3.a) for intra-scanner and inter-scanner settings (see Figure 3 and 4). Specifically, in the inter-scanner setting, icobrain-dl outperformed icobrain-nondl and FastSurfer, except in the right white matter and left cortical gray matter. Notably, icobrain-dl produced significantly lower inter-scanner test-retest errors ($p < 0.01$) across all substructures, including the caudate nucleus, hippocampus, globus pallidus, putamen, and thalamus.

### Diagnostic performance

The performance of icobrain-dl in detecting pediatric patients with CVI surpassed childmetrix (AUC of 0.48) and FastSurfer (AUC of 0.60), with an AUC of 0.69, as shown in Table 4. There was no statistically significant difference between icobrain-dl and FastSurfer in terms of AUC. Nevertheless, icobrain-dl exhibited significantly superior performance compared to childmetrix ($p < 0.05$).

**Table 4:** The proposed method has superior performance in detecting pediatric patients with Cerebral Visual Impairment (CVI) from those without CVI using the white matter volume normalized for head size (dataset 4.p) and comparable high performance in detecting adult patients with Alzheimer’s Disease from age-matched controls using the cortical grey matter of the temporal lobe normalized for head size (MIRIAD, dataset 4.a).

	Pediatric dataset 4.p			Adult dataset 4.a		
	icobrain-dl	childmetrix	FastSurfer	icobrain-dl	icobrain-nondl	FastSurfer
AUC	0.69	0.48	0.60	0.99	0.98	0.98
Accuracy	0.71	0.57	0.67	0.96	0.93	0.96
Specificity	0.86	0.57	0.86	0.91	0.91	0.96
Sensitivity	0.64	0.57	0.57	1	0.96	0.96

*AUC* = area under the curve.The AUC obtained by icobrain-dl was significantly higher than the AUC obtained by childmetrix ($p < 0.05$) while there was no statistically significant difference between icobrain-dl and FastSurfer, and icobrain-dl and icobrain v5.9 using the DeLong test. The accuracy, specificity, and sensitivity metrics are assessed at the maximum value of the Youden index.

In supporting the classification of AD patients from age-matched controls, the icobrain-dl demonstrated comparable high performance in terms of accuracy, sensitivity, and specificity. The AUC for icobrain-dl was 0.99, icobrain-nondl was 0.98, and FastSurfer was 0.98, with no statistically significant difference.

### Computational time

On average, the proposed method took approximately 5 minutes to complete the entire pipeline when running on a server without a GPU (amazon web services cloud environment c6i.2xlarge, 8vCPU and 16GiB of Memory RAM) while the pipeline based on FastSurfer requires nearly 6 minutes on a GPU server (cloud environment p2.xlarge, NVIDIA Tesla K80 (12 GiB), 4vCPU and 61GiB of Memory RAM). In contrast, the non-deep learning approaches childmetrix and icobrain v5.9 running on a server without a GPU (cloud environment c6i.2xlarge, 8vCPU and 16GiB of Memory RAM) required on average 24 minutes and 27 minutes.

### Qualitative results

**Figure 5:**
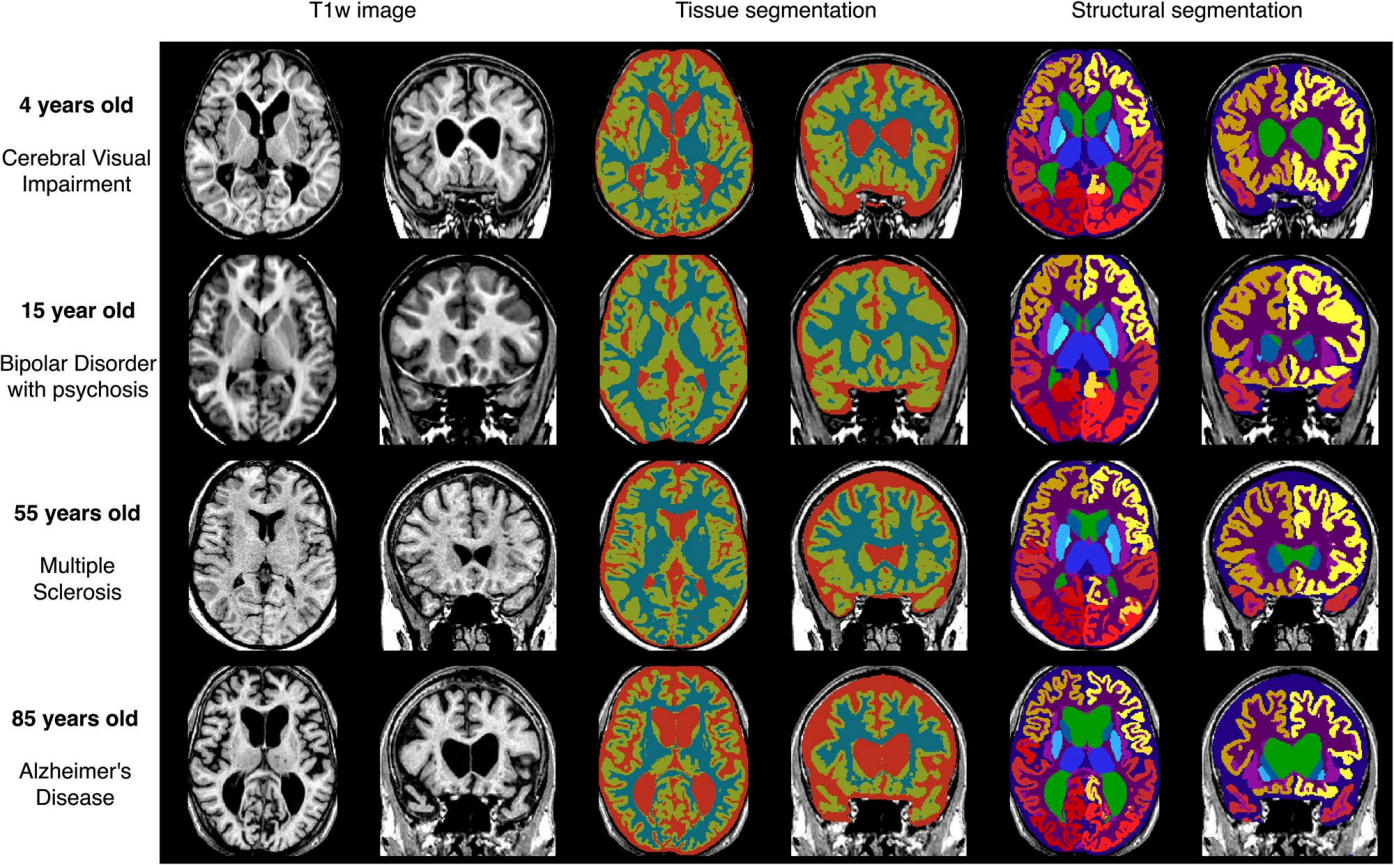
Examples of segmentations of icobrain-dl on test patients with different ages and pathologies. The pipeline accurately quantifies brain tissues and structures despite variations in age, pathology, and intensity contrast, capturing anatomical variability such as the cortical atrophy patterns characteristic of patients with Alzheimer’s Disease.

Figure 5 illustrates the segmentation results of icobrain-dl in test patients across the lifespan, with ages ranging from 4 to 85 years old. These qualitative results demonstrate the model’s robustness to diverse pathological conditions and scans with differing intensities and contrasts.

## Discussion

This study introduces icobrain-dl, a deep learning-based pipeline capable of performing quantitative assessment of brain tissues and structures across pediatric and adult populations.

The pipeline was developed and validated using T1-weighted images obtained from various scan vendors with different magnetic field strengths. The dataset includes patients across a broad age range with various pathological conditions. Evaluation of the proposed pipeline included segmentation accuracy and reproducibility assessments, along with an exploration of its clinical application through diagnostic performance and computational efficiency.

In contrast to methods tailored for specific age ranges, such as childmetrix for children or icobrain-nondl and FastSurfer for adults, icobrain-dl provides quantitative brain measurements across the human lifespan, from early childhood (i.e., 4 years old) to maturation and older age, within a single deep learning model. Previous experiments have shown the accuracy performance of adult-trained models in pediatric data^9,10^. However, in this study, we explicitly included pediatric data to train the model and observed that it does not compromise the performance on scans from adult subjects, and vice versa. Furthermore, the inclusion of a pediatric cohort allowed the deep learning model to learn and adapt to challenges associated with brain development, including reduced tissue contrast, within-tissue intensity heterogeneities, and smaller regions of interest. The proposed single deep learning model eliminates the need for multiple age-specific segmentation models, enabling consistent measurements across transitional phases, such as from the pediatric stage through adolescence to adulthood. This facilitates the creation of a reference standard for human brain development, essential for quantifying developmental changes, interpreting deviations, and identifying patterns of anatomical differences in neurological and psychiatric disorders that manifest during various stages of development and aging^34^.

High reproducibility is crucial for accurately measuring brain changes and atrophy^35^. The proposed icobrain-dl, was compared with state-of-the-art brain segmentation models, including childmetrix^15^, FastSurfer^11^ and the medical device software icobrain-nondl (i.e., icobrain v5.9^7,8^). The results demonstrated overall superior reproducibility assessed in pediatric intra-scanner and adult intra- and inter-scanner scenarios, particularly in the adult inter-scanner setting, with significantly lower variability observed in all brain substructures (p < 0.01). This improvement can be attributed to the diverse sources of T1-weighted images used in training, along with the integration of a data augmentation algorithm. This algorithm enhanced the variability of training data in terms of intensity and contrast, which has been proven to be particularly beneficial for repetitions in different scanners (i.e., inter-scanner)^29^.

Volumetric imaging biomarkers provided by icobrain-dl required good accuracy, specificity and sensitivity to be used as a metric for diagnosis (e.g., distinguishing patients with Alzheimer’s vs. healthy controls). The proposed pipeline exhibited comparable diagnostic performance to state-of-the-art methods, achieving the highest AUC for both clinical conditions. It is important to note that the purpose of the diagnostic performance scenario was to compare different methods using the same measurement, rather than to identify clinically relevant imaging biomarkers for specific pathologies. Future studies will explore the potential of volumetric imaging biomarkers to enhance our understanding of the underlying mechanisms of diseases and improve their diagnosis, particularly in complex and partly understood conditions like CVI. This involves increasing sample sizes and considering factors such as sexual dimorphism^36^ and age-dependent developmental trajectories^13^.

The proposed pipeline also analyses the images faster than traditional segmentation approaches, aligning with findings from previous studies employing deep learning models^9,11^. However, in contrast with previous deep learning models, the proposed model deployed a lightweight deep learning architecture, consisting of relatively few layers. This design choice aimed to reduce the computational complexity, facilitating model inference on CPU-only platforms and ensuring efficient segmentation without incurring the elevated economical costs associated with GPU usage. The reduced processing time avoids creating additional bottlenecks in the radiological workflow.

The annotation protocols used to establish the ground truth of brain structures may vary across datasets, potentially differing from our definition of brain structure borders. This discrepancy could explain the higher overlap observed between models than the overlap between models and ground truth. Notably, icobrain-dl and the age-specific models are trained on datasets with overlapping patients and employ the same annotation protocol.

The icobrain-dl pipeline is designed to use T1-weighted images to analyse the structural anatomy of the brain. Currently, its application is limited to conditions characterized by non-mass effects due to the absence of multimodal data, such as fluid-attenuated inversion recovery (FLAIR) images. However, future iterations of icobrain-dl aim to integrate multimodal data, thereby expanding its utility to cover a broader spectrum of pathologies.

The proposed deep learning model covers the human lifespan, starting at 4 years of age. The period preceding this age is the most dynamic phase of postnatal human brain development^37^. Maturation processes, including myelination, notably influence T1-weighted image contrasts, for instance, shifting from hypointense white matter in newborns to hyperintese in 2-year-old infants, making the development of a reliable segmentation model a very complex task. Hence, additional exploration is required to incorporate quantification of brain segmentation during this initial phase of brain development.

## Conclusion

The proposed deep learning-based pipeline, icobrain-dl, is capable of quantifying brain tissues and structures across the human lifespan beginning at 4 years of age. Extensive validation in clinically relevant settings has demonstrated its ability to provide accurate and reproducible volume quantification of relevant brain anatomical structures from T1-weighted images.

By offering a unified solution from early childhood to maturation and older age, icobrain-dl has the potential to significantly enhance research and clinical applications in monitoring brain development and diagnosing neurological conditions.

### Supplementary Information

Supplementary Information.

## Data Availability

Further details regarding the publicly available datasets analyzed in the current study can be found in Appendix A. Additional datasets analyzed during the current study can be made available from the corresponding author with the permission of a third party upon reasonable request. The code employed in this study is not publicly accessible due to commercial restrictions but is available from the corresponding author upon reasonable request.
