# Supplementary material for: A deep learning model for brain segmentation across pediatric and adult populations
Source: Sci Rep. 2024 May 22;14:11735. doi: 10.1038/s41598-024-61798-6 (PMC11111768; doi:10.1038/s41598-024-61798-6)
Supplement: Supplementary file 1 — Supplementary Information. [file 41598_2024_61798_MOESM1_ESM.pdf]

Supplementary Material: A deep learning model for brain segmentation across pediatric and adult populations

Appendix A. Datasets

The publicly available datasets used in this study are:

Dataset 1.1.p is available at: http://fcon_1000.projects.nitrc.org/indi/cmi_healthy_brain_network/.

Dataset 1.2.p, which received support from the Canadian Institutes of Health Research (grant numbers IHD-134090, MOP-136797) and the Alberta Children's Hospital Research Institute, is available at: https://osf.io/axz5r/.

Dataset 2.p is available at: https://www.nitrc.org/projects/candi_share/.

Dataset 2.a is available in open access by OASIS (https://www.oasis-brains.org/) and manual labelings by Neuromorphometrics, Inc. (http://Neuromorphometrics.com/) under academic subscription. The data is released under the Creative Commons Attribution-NonCommercial license (CC BY-NC) with no end date.

Dataset 3.p is available at: https://fcon_1000.projects.nitrc.org/indi/CoRR/html/nki_2.html.

Dataset 4.a was obtained from the MIRIAD database (https://www.ucl.ac.uk/drc/research/research-methods/minimal-interval-resonance-imaging-alzheimers-disease-miriad.). The MIRIAD investigators did not participate in analysis or writing of this report. The MIRIAD dataset is made available through the support of the UK Alzheimer's Society (Grant RF116). The original data collection was funded through an unrestricted educational grant from GlaxoSmithKline (Grant 6GKC).

Figure S.1 and S.2 illustrate the age distribution of the datasets used in this study.

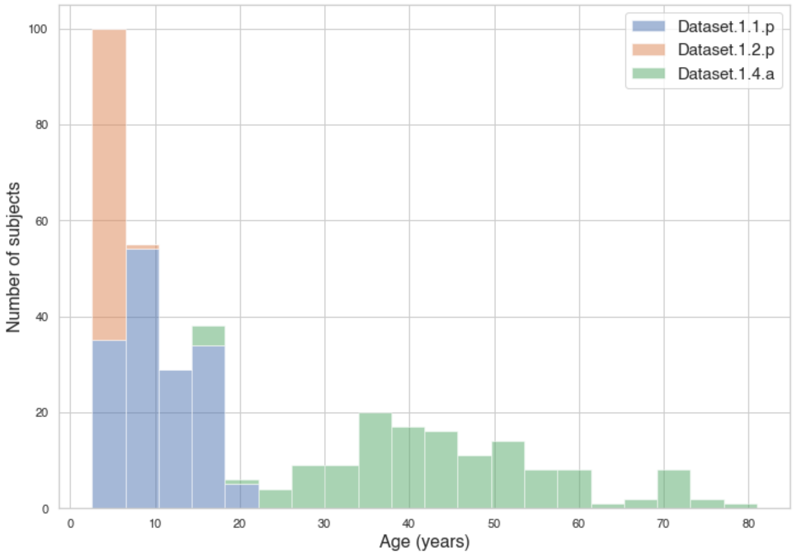

Figure S.1. Age distribution in the training datasets. Dataset 1.3.a is not included

.

Appendix B. Brain structures segmented by icobrain-dl

1. Background
2. WM background
3. GM background
4. CSF background
5. Thalamus
6. Hippocampus
7. Putamen

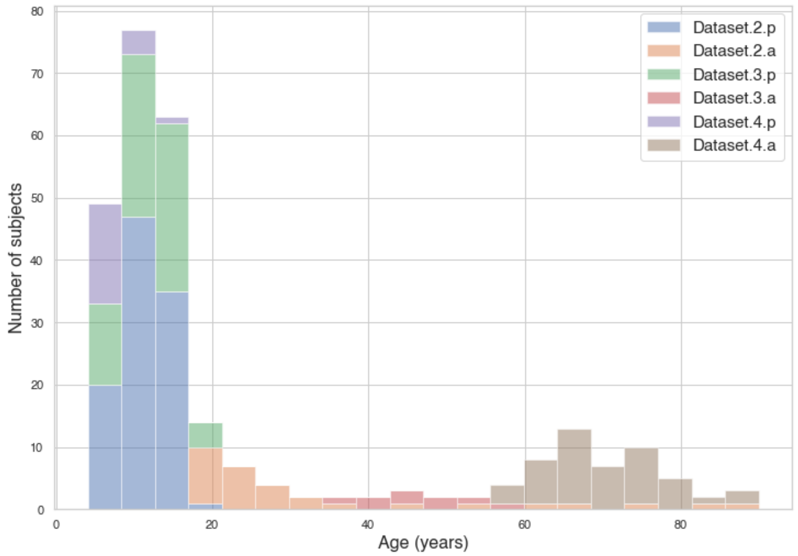

Figure S.2. Age distribution in the validation datasets

.

8. Caudate
9. Pallidum
10. Midbrain
11. Pons
12. Medulla
13. Cerebellum
14. Upper lateral ventricles
15. Inferior lateral ventricles
16. Amygdala
17. Left CGM Frontal lobe
18. Right CGM Frontal lobe
19. Left CGM Parietal lobe
20. Right CGM Parietal lobe
21. Left CGM Occipital lobe
22. Right CGM Occipital lobe
23. CGM Temporal lobe

Additional structures can be easily derived from these initial segmentations. For example, the cerebellar gray matter is computed as the intersection of gray matter (task 1) and cerebellum (task 2).

Appendix C. Multi-task architecture

The proposed model, icobrain-dl uses a two-headed U-net as backbone, i.e., it produces two predicted segmentation masks to classify voxels in terms of brain tissues and structures, respectively. In principle, a single output mask could be sufficient if each voxel received the most fine-grained label available. For example, at the location of the *hippocampus*, voxels could have the label *GM* for task 1 and the label *hippocampus* for task 2. Since prior anatomical knowledge allows inferring the *GM* label from the *hippocampus* label, the second label can be considered sufficient.

However, we argue that employing both a "coarse-grained" and a "fine-grained" segmentation in separate tasks offers several advantages:

- Task 1 has a simpler (i.e., easier to learn) label structure, facilitating the model's training. In the initial stages of training, this helps to guide the model to acquire meaningful representations that encode at least the three main tissue classes. Essentially, task 1 serves as a regularizer for task 2, facilitating faster convergence and more effective learning.
- By using two tasks with their respective loss functions, more detailed model evaluation and debugging can be performed during and after training. For instance, a very low loss on task 1 but a high loss on task 2, is a clue that class mixing between different GM structures is the limiting factor.
- After training, the output of task 1 can be utilized for post-processing or correcting the output of task 2.

In summary, the adoption of a multi-task setup with a "coarse" (i.e. tissue) and a "fine-grained" (i.e. structural) segmentation offers benefits such as improved model training, detailed evaluation and debugging capabilities, and the potential for post-processing and correction. These advantages contribute to the overall effectiveness and reliability of the segmentation model.
